# Associations between HEXACO personality traits, substance use disorders, and behavioral addictions: a protocol for a comprehensive systematic review and meta-analysis

**DOI:** 10.1186/s13643-024-02741-8

**Published:** 2025-01-02

**Authors:** Farangis Sharifibastan, Eilin Kristine Erevik, Katharina Teresa Enehaug Morken, Ståle Pallesen

**Affiliations:** 1 Department of Psychosocial Science, University of Bergen, Bergen, Norway; 2 Norwegian Competence Center for Gambling and Gaming Research, University of Bergen, Bergen, Norway; 3 Department of Clinical Psychology, University of Bergen, Bergen, Norway; 4 Department of Addiction Medicine, Haukeland University Hospital, Bergen, Norway

**Keywords:** HEXACO model, Personality traits, Substance use disorders, Behavioral addictions

## Abstract

**Background:**

While research has explored the connection between addiction and personality, no systematic study has examined how substance use disorders (SUD) and behavioral addictions specifically relate to the HEXACO model of personality. This systematic review and meta-analysis aim to fill this gap by investigating the association between HEXACO personality traits and various addictions, including illegal substances (e.g., narcotics and cannabis) and behavioral addictions (e.g., gambling, gaming, social media addiction, and compulsive sexual behavior disorder) across different populations.

**Methods:**

The protocol is in accordance with the Preferred Reporting Items for Systematic Review and Meta-Analysis Protocols (PRISMA-P) statement. Searches will be conducted in databases including APA PsycINFO (Ovid), MEDLINE (Ovid), ProQuest, Web of Science, CINAHL, Wiley Online Library, and Google Scholar. Empirical studies published as full papers in peer-reviewed journals or as full dissertations, in English, other European languages, or Persian, investigating the association between HEXACO personality traits and addictive disorders are eligible. Two reviewers will independently screen all citations and full-text articles, and extract data using the Covidence software. They will further assess the risk of bias and quality of the studies using the Newcastle–Ottawa Scale (NOS) for longitudinal and cohort studies, an adapted version of the NOS for cross-sectional studies. Publication bias will be evaluated using funnel plots, Egger’s test, and trim and fill analysis. In addition to a narrative summary, meta-analyses will be conducted if data are sufficient. Random effects models will be used to pool effect sizes. Subgroup analyses and meta-regression will be performed to investigate potential sources of heterogeneity. Sensitivity analyses will examine the robustness of the results. Data analysis will be conducted using Comprehensive Meta-Analysis (CMA), version 4.

**Discussion:**

This review and meta-analysis will be the first to systematically explore and integrate the evidence available on the association between the HEXACO personality traits and SUD and behavioral addictions. By consolidating information, the study will enhance our understanding of the role of personality traits in the development, maintenance, and treatment of SUD and behavioral addictions.

**Systematic review registration:**

PROSPERO CRD42023468153.

**Supplementary Information:**

The online version contains supplementary material available at 10.1186/s13643-024-02741-8.

## Background

Addictions pose a great threat to public health, encompassing both substance use disorders (SUDs) and behavioral addictions. SUDs involve excessive and uncontrolled consumption of psychoactive chemicals such as alcohol or other psychoactive substances affecting about 35 million people worldwide [1, 2]. The impact of SUDs is profound, with the 2017 Global Burden of Disease study ranking them as the second leading cause of disability among mental disorders, accounting for 31,052,000 (25%) years lived with disability (YLD) [3, 4].

The scope of addiction extends beyond SUDs to include behavioral addictions. According to official psychiatric diagnostic systems, there are currently only two non-chemical addictions recognized (i.e., pathological gambling and gaming disorder) [5, 6]. However, many scholars have argued and found empirical support for various other non-chemical/behavioral addictions [7, 8], including social media addiction [9], video game addiction [10], Internet addiction [11], exercise addiction [12], mobile phone addiction [13], shopping addiction [14], workaholism [9], and sex addiction [15, 16]. Many people are affected by behavioral addictions, with an estimated weighted average prevalence of 2.47% for internet gaming disorder (IGD) [2, 17], and 4.5% for pathological gambling disorder (PGD) [2, 18].

At a broad level, studies indicate that addiction represents a multifaceted challenge, impacting not only individual health but also society at large [19]. This issue has detrimental effects on both mental and physical well-being, leading to significant negative outcomes [20]. Unfortunately, psychologists and psychiatrists are less involved with addictions, both theoretically and therapeutically, than they are with other mental disorders [21]. Several dispositional factors have been found to increase the likelihood of engaging in a variety of potentially addictive behaviors, both recreationally and at a problematic level [22], one of which is personality traits. In most studies examining associations between personality traits and addiction disorders, some traits are considered predisposing factors for addiction [23]. Therefore, certain personality characteristics may represent independent risk factors for addiction. The Five-Factor Model (FFM) is a predominant framework used to conceptualize personality traits. This model consists of five overarching domains that capture fundamental aspects of human personality. Extraversion (vs. introversion), agreeableness (vs. antagonism), conscientiousness (vs. independently or disinhibition), neuroticism (vs. emotional stability), and openness to experience (vs. closeness) are the higher-order domains in this model [24, 25]. The FFM`s personality trait profiles associated with several clinical disorders have been examined, including alcohol use disorder (AUD], SUD [26], gambling disorder (GD] [27], Instagram addiction [28], smartphone addiction [29], social media addiction [30], online game addiction [31], and sex addiction [32, 33]. Findings from these studies indicate that the relationships with personality traits vary among different addictions, a factor that could hold significance for both theoretical understanding and practical applications [34]. Several meta-analyses have examined the FFM in relation to alcohol and substance abuse, finding that conscientiousness, and agreeableness are inversely associated with most addictions. However, neuroticism tends to be positively associated with these addiction disorders. However, conscientiousness tends to be inversely associated with these addiction disorders [35, 36]. There has been a lack of consistency in the results regarding extraversion and openness and substance addictions.

The FFM is widely adopted in the literature on addiction, however, other research has identified alternative personality structures using a similar lexical methodology [37]. It has been claimed and illustrated empirically that certain aspects of personality might be underrepresented in the FFM (for example, Honesty-Humility) [38]. As an alternative to the FFM, Lee, and Ashton [39] present the HEXACO model of personality to address some of the limitations of the FFM and to provide an expanded method of examining personality characteristics. This model was developed from lexical studies that involved self and observer ratings of personality descriptive adjectives across various languages [40, 41]. These studies indicated a six-factor solution to describe the variation in personality. According to the HEXACO model, the six main dimensions are Honesty-Humility (H), Emotionality (E), Extraversion (X), Agreeableness (A), Conscientiousness (C), and Openness to Experience (O). While the extraversion, conscientiousness, and openness to experience dimensions in the HEXACO model largely align with their counterparts in the FFM model [42], the HEXACO model introduces rotated variants of agreeableness and emotionality [39]. HEXACO model, for example, attributes traits like even temper, which reflects low neuroticism in the FFM, to agreeableness. Conversely, traits such as sentimentality, which are typically associated with agreeableness in the FFM, are assigned to emotionality. The most significant deviation from the FFM is the inclusion of honesty-humility as a sixth fundamental dimension of personality. Numerous studies have provided evidence supporting the validity of the honesty-humility factor [43–47]. This factor represents the sincerity of an individual regarding their interactions with others, the willingness to take advantage of others for personal gain, the desire for or motivation to acquire high status, as well as the modesty of the individual [48]. There have been numerous studies examining honesty-humility in various contexts, including those related to gambling severity [49–51], SUD [52], the workplace [53], academic settings [48], and laboratory studies examining decision-making in social dilemmas [54]. The research landscape exploring the relationship between the HEXACO model of personality, SUD, and behavioral addictions is evolving, with a growing number of studies emerging. Abbasi et al. (2014) investigated the relationship between personality traits, based on the HEXACO model, and cell phone addiction and found a significant positive relationship between cell phone addiction and emotionality, as well as a significant inverse relationship between cell phone addiction, extraversion, and conscientiousness [55]. Enayati (2020) found that mobile phone addiction had a significant inverse relationship with honesty-humility, extraversion, conscientiousness, openness to experience, and a positive correlation with emotionality [56]. Azizi et al. (2015) studied the associations between internet addiction and the HEXACO model of personality traits. They found that among the HEXACO personality traits, only extraversion and openness to experience were significantly and positively related to internet addiction [57]. Horwood and Anglim (2018) studied a sample of Australian adults and found a moderate negative association between problematic smartphone use and honesty-humility, agreeableness, conscientiousness, and openness to experience. They also found a significant positive correlation between problematic smartphone use and emotionality [58]. Inanloo et al. [59] examined a model predicting online game addiction based on the HEXACO personality traits and the parent–child relationship, mediated by impulsiveness. They found that the direct path coefficients of conscientiousness, honesty-humility, extraversion, agreeableness, and openness to experience on online game addiction were not significant. Zafar et al. (2018) investigated the relationship between Facebook addiction and HEXACO personality traits among university students and found significant positive correlations between Facebook addiction and extraversion, emotionality, and openness to experience, while honesty-humility, agreeableness, and conscientiousness displayed significant negative correlations [60]. Leslie and McGrath (2023) used the HEXACO model to compare personality traits of gamblers who played exclusively online, exclusively offline, and in mixed-mode contexts and found that mixed-mode gamblers reported lower honesty-humility scores and higher extraversion scores compared to exclusively online and offline gamblers [50]. McGrath et al. (2018) investigated the relationships between personality traits, based on the HEXACO model of personality, and problem gambling severity in young adult gamblers. They found that honesty–humility, agreeableness, and conscientiousness were significantly and negatively associated with the scores on the Problem Gambling Severity Index (PGSI) [51]. Kim et al. (2018) studied the associations between HEXACO traits and problem gambling severity in a community-recruited sample of gamblers. They found that scores on honesty–humility, conscientiousness, and openness to experience were significantly and inversely associated with gambling severity [49]. Rash et al. (2018) examined HEXACO personality traits in relation to disordered engagement in three addictive behaviours: AUD, cannabis use disorder (CUD), and GD. Multinomial logistic regression analyses revealed lower levels of honesty-humility among individuals with AUD and GD, and higher levels of openness to experience among individuals with CUD compared to control participants [52].

Taken together, the literature on the relationship between the HEXACO personality traits, SUD, and behavioral addictions consistently shows that honesty-humility [49–51, 58, 60], agreeableness [51, 58, 60, 61], and conscientiousness [51, 55, 56, 58, 60] are inversely associated with most addictions. In contrast, the trait of emotionality is positively associated [55, 56, 58, 60]. Extraversion [50, 57, 60] and openness to experience [52, 57, 60] have been shown to positively relate to behavioral addictions in some studies, however, in other studies, extraversion [55, 56] and openness to experience [31, 56, 58] have been shown to be inversely related to addictive disorders. As research about the relationship between HEXACO personality traits, SUD, and behavioral addictions expands, it is necessary to synthesize and summarize these findings to draw conclusions. One rigorous method for aggregating numerical research findings is meta-analysis. Meta-analysis refers to a collection of statistical methods that are used to combine the results of independent experimental and correlational studies leading to an overall estimate or result [62]. To date, no meta-analysis has been conducted investigating the relationship between HEXACO personality traits, SUD, and behavioral addiction. This gap in the literature necessitates a project that systematically reviews and synthesizes existing findings. Conducting this study is essential for four main reasons. Firstly, individual studies have assessed the association between HEXACO traits and various types of addiction, but their results have been inconsistent. Secondly, a systematic review and meta-analysis can provide a more robust and comprehensive understanding of these relationships by aggregating and analyzing data from multiple studies. Thirdly, knowing which HEXACO personality traits are associated with SUD and behavioral addictions could provide insight into potential mechanisms that contribute to addiction which could further inform future research and intervention strategies. Fourthly, identifying relevant moderators concerning the relationship between the HEXACO model and addictions will aid in identifying circumstances where the personality traits in question may play an important role in developing addictions.

## Scope and objectives of the present study

This systematic review and meta-analysis aim to comprehensively examine the associations between HEXACO personality traits, SUD, and behavioral addictions. The main scope of the study is to include all studies investigating the term “HEXACO” and addiction disorders including illegal substances, and behavioral addictions (e.g., gambling and gaming problems, social media addiction, and CSBD). The study includes diverse populations, covering both clinical and non-clinical samples across various age groups (above 18 years), genders, socio-economic backgrounds, and ethnic identities. By including studies from any sample type and geographical location published in English, other European languages, or Persian, we ensure a broad and inclusive scope. This systematic review involves a meta-analysis of all relevant studies and will report effect sizes converted into correlation coefficients to standardize the measurement of associations across studies. Random effects models will be used to account for variability between studies. Additionally, we will assess several moderating factors in the relationship between HEXACO personality traits, SUD, and behavioral addictions. Following the recommendation to include approximately 10 studies for each covariate when investigating potential moderators through meta-regression analyses [62], the number of variables that will be entered into the meta-regression models will depend on the number of studies that will be included in the specific analysis. The research team will select a priori and in prioritized order to test (a) mean age, (b) gender, (c) ethnicity, (d) marital status, (e) occupational status, and (f) educational attainment.

We aim to answer the questions (1) Is there an association between HEXACO personality traits and the use of illegal substances, including alcohol, nicotine, and all narcotics and cannabis? (2) Is there an association between HEXACO personality traits and behavioral addictions, including gambling and gaming problems, social media addiction, and CSBD? and (3) Are associations between HEXACO personality traits moderated by age, gender, ethnicity, marital status, occupational status, and educational attainment?

## Methods/design

This protocol has been registered within the International Prospective Register of Systematic Reviews (PROSPERO; registration number: CRD42023468153) [63] and was reported according to the PRISMA for Protocol (PRISMA-P) 2015 statement [64] [see Additional file 1].

### Eligibility criteria

For studies to be eligible for inclusion, they will have to meet the following criteria: (1) being an empirical study; (2) original articles, published as full papers; (3) published in English or other European languages or Persian; (4) published in a peer-reviewed journal; (5) master’s or doctoral dissertations, published as full dissertation; (6) studies investigating the association between HEXACO personality traits and addictive disorders, including illegal substances (e.g., all narcotics and cannabis as separate categories), and behavioural addictions (e.g., gambling and gaming problems, social media addiction, and CSBD); (7) research reporting Pearson’s or Spearman’s r correlation coefficients of the variables of interest, or any data that could be converted into a correlation coefficient, such as Cohen’s *d*/*f*, *T* value, or Fishers *Z*; (8) studies with any population type (clinical or non-clinical), interventions, and participant characteristics; and (9) with any year of publication. Studies will be excluded if they are (1) based on case studies and qualitative designs; (2) articles not published in a peer-reviewed journal; (3) grey literature, including conference papers, reports, newspaper articles, and unpublished dissertations (to ensure the inclusion of high-quality, rigorously vetted studies); (4) studies without full-text access; (5) measuring personality traits similar to the HEXACO model, but not the actual traits; (6) not related to SUD or behavioral addiction; (7) not report sufficient data for calculating the effect size (Pearson’s or Spearman’s *r* correlation coefficients of the variables of interest, or any data that could be converted into a correlation coefficient); and (8) articles or dissertations that have been identically presented to two different journals or universities under different titles (Only the most recent or comprehensive (with highest number of observations) version of a duplicate will be included to avoid redundancy).

### Information sources

Literature searches will be conducted across various electronic literature databases, such as APA PsycINFO (Ovid), MEDLINE (Ovid), ProQuest, Web of Science, PubMed, CINAHL, and Wiley Online Library. Also, a literature search will be conducted on Google Scholar, of which we will review the first 50 pages due to a large number of results (over 12,000 hits).

### Search strategy

We chose “HEXACO” as our one and only search term, after advice from a research librarian. In the case of the HEXACO model, there is a limited number of published articles. The HEXACO model of personality is a new phenomenon with limited existing evidence. Searching with the singular search term “HEXACO”, we assume that we are guaranteed to identify all articles on HEXACO. Making a more elaborate combination of search terms will necessarily limit the number of search results, and we could potentially miss out on relevant articles.

Being a relatively new phenomenon explains why “HEXACO” is not yet an established term in Controlled Vocabulary/Subject Headings. Including the more general Subject Headings of “Personality traits” or “Personality Tests” in the search strategy would include a very large number of articles not relevant to the research question of this review.

No applied limits will be used in the search strategy, and the reason for this is the same as the rationale for using only the one search term “HEXACO”: The safest way of ensuring that no relevant articles are missed, is to do all excluding of articles in the screening process. This is possible to do because of the fact that the one search term “HEXACO” with no limits applied results in a manageable number of results to screen and this approach was recommended by a scientific librarian. The search field definition chosen in each database will be the default alternative, covering a minimum of titles and abstracts. As an example, our APA PsycINFO (Ovid) Search Strategy is as follows: hexaco.mp. [mp = title, abstract, heading word, table of contents, key concepts, original title, tests and measures, mesh word].

In addition to searching with the term “HEXACO” with the default search field definition in all the mentioned databases, we will employ additional methods in our literature search. We will employ backward tracking (inspecting reference lists in relevant identified literature). Moreover, we leverage artificial intelligence in our research, specifically using Keenious Plus by transferring relevant articles into its platform to identify matching articles that are relevant to our research, helping us uncover any potentially missed articles.

### Selection process

An initial screening of titles and abstracts of identified papers will be performed by two independent reviewers (FS and PH), who will assess each study for relevance according to the pre-specified eligibility criteria. Studies that cannot be conclusively excluded from the title and abstract screening will be taken forward to full-text screening, at which stage the full text will be obtained and a second screening process performed, again by two independent reviewers. This will result in a final set of papers to be included in the review. Discrepancies between the two reviewers at any stage will be resolved through discussion and, if required, referral to a third reviewer (EKE). The number of studies identified, included, and excluded at each stage will be reported using the PRISMA flow diagram [65] together with reasons for exclusion at the full-text stage. Moreover, to calculate interrater reliability and the degree of agreement between the initial choices made by two independent raters, we will use Cohen’s kappa statistic or percentage agreement [66].

The pilot screening process, involving 20 studies, was conducted to ensure the consistency and reliability of our methods before full-scale screening. Reviewers (FS and PH) independently screened these articles for inclusion based on predefined inclusion criteria. There was a high level of agreement among the reviewers in the screening process, with a Cohen’s kappa coefficient of 0.89, indicating substantial agreement. Differences primarily arose in the application of inclusion criteria at the initial stage. These differences were resolved through discussions among the reviewers, leading to minor modifications in the inclusion criteria for clearer guidance.

### Data management

Covidence Systematic Review software (69) will be used to manage references throughout the review process. Covidence’s systematic approach ensures an organized and efficient review, enhancing both reliability and collaboration between reviewers. This comprehensive software will be employed at all stages, including screening titles and abstracts, reviewing full texts, and extracting data. Initially, all relevant articles identified from the database searches will be imported into Covidence. The software will then automatically remove duplicates, after which two independent reviewers will commence the screening process.

### Data extraction

The Covidence Systematic Review software allows us to incorporate personalized headings, subheadings, single-choice fields, multiple-choice, and tables [67]. A meticulous data extraction sheet will be developed based on our study objectives. This sheet will be made available within the Covidence platform, and accessible to the reviewers responsible for data extraction. This development will be guided by the outcomes of the full-text screening stage, following templates provided by experts in systematic reviewing within the platform. Two independent reviewers (FS and PH) will conduct data extraction in this platform for all included studies. In instances where multiple publications arise from a single study, we will consolidate all sources and extract the data into a unified form. The data items to be extracted encompass various elements, including study design, age and gender of participants, country of study, sample size (*n*), setting, methods of assessing personality traits, SUD, and behavioral addictions. Additionally, relevant findings, including effect sizes converted to correlation coefficients (*r*), will be extracted. If the correlation coefficients are not provided directly, we will also extract sample size, standard deviation (SD), and means for both the addiction and control groups to facilitate the conversion to the correlation coefficients. The list of variables to be extracted is shown in an additional file [see Additional file 2].

The pilot test for data extraction, involving seven studies, was conducted to assess the feasibility of the data extraction process and ensure consistency among reviewers. Reviewers (FS and PH) independently extracted data from these studies using a predefined data extraction form. There was a high level of agreement among reviewers, with a Cohen’s kappa coefficient of 0.85, indicating substantial agreement. Discrepancies were resolved through discussion among the reviewers, resulting in minor modifications to the data extraction form to clarify ambiguous conditions. This pilot confirmed the feasibility and robustness of our data extraction process for the upcoming comprehensive meta-analysis.

### Quality and risk of bias assessment

Assessing the risk of bias in included studies is important in determining the validity of the results and interpretation of findings. The Newcastle–Ottawa Scale (NOS) for longitudinal and cohort studies, and an adapted version of the NOS for cross-sectional studies, will be used to assess the risk of bias and quality of the studies [68, 69]. The NOS provides predefined criteria for assessing bias/quality through a checklist consisting of three main categories. It is possible to obtain a maximum score of 10 stars, where a higher score indicates higher quality/less bias. The first category is “selection” and relates to the representativeness of the sample, sample size, comparability between respondents and non-respondents, and ascertainment of the exposure. This category provides a maximum of five stars. The second category is “comparability” and concerns whether confounding factors are controlled for. This category provides a maximum of two stars. The third category is “outcome” and represents the assessment of the outcome and the statistical tests. The maximum score in this category is three stars. In the current study, studies with five stars or more will be considered to have moderate to good quality.

### Data analysis and synthesis

This study will report effect sizes that will be converted into correlation coefficients to standardize the measurement of associations across studies. We plan to combine data from studies with different designs in a single meta-analysis, as long as they provide comparable effect size estimates. This approach allows us to synthesize a broad range of evidence, enhancing the generalizability of our findings.

We will use random effects models to pool effect sizes rather than fixed effects models due to the anticipated variations in sample characteristics, methods, and measures across the included studies. Random effects models account for between-study heterogeneity and provide more conservative and generalizable estimates when there is underlying true variance in effect sizes [70]. Specifically, we will use the DerSimonian-Laird method for the random effects model, which is a widely used approach for meta-analysis that estimates the between-study variance and weights the studies accordingly. This approach is particularly suited for our research given the diverse nature of addictions and personality dimensions being investigated.

To assess the heterogeneity among studies, Cochran’s *Q* test and the *I*^2^-index will be employed. We will interpret the *I*^2^-values as an indication of the proportion of total variation in estimated effects that may be attributed to heterogeneity rather than to chance. An *I*^2^-value greater than 50% will be indicative of substantial heterogeneity [71]. As the correlation coefficient is not normally distributed, we will use *Z*-transformation $Z=\frac{1}{2}\text{ln (}\frac{1+\rho }{1-\rho })$ in the meta-analytic calculations. Meta-analysis will be used to pool the results of studies.

To investigate the impact of publication bias, which can occur when the likelihood of a study being published is influenced by the nature or direction of its results, we will construct funnel plots and conduct regression-based Egger’s test and non-parametric Trim and Fill analysis. These analyses will help identify and adjust for asymmetry in the effect size distribution that may suggest the presence of publication bias.

Subgroup analysis and meta-regression will be conducted to explore potential sources of heterogeneity. Subgroup analysis will be performed according to age, gender, ethnicity, marital status, occupational status, and educational attainment. The specific criteria for defining these subgroups will be determined after a comprehensive review of the included studies, ensuring clarity and appropriateness for each variable. This approach aims to enhance our understanding of how these variables may differentially impact the association between HEXACO personality traits and addiction-related outcomes.

Sensitivity analysis will be performed using studies based on representative samples to assess how the exclusion of studies of potentially lower methodological rigor or with specific population characteristics affects the robustness of our findings. This analysis will help ensure the conclusions drawn from our meta-analysis are not unduly influenced by a subset of the included studies. Data will be analyzed with CMA VER.4 [72].

## Discussion

To the best of our knowledge, this systematic review and meta-analysis will be the first to systematically assess the association between the HEXACO personality traits and addiction disorders, including illegal substances (e.g., all narcotics in one category and cannabis in another), and behavioral addictions (e.g., gambling and gaming problems, social media addiction, and CSBD), within all types of sample populations.

The findings of this systematic review and meta-analysis have several important implications for both research and clinical practice in the field of addiction. From a research perspective, this study can improve theoretical models of addiction by including the HEXACO framework and revealing research gaps. This comprehensive approach may provide a more nuanced understanding of the complex interplay between personality traits and addiction. In terms of clinical applications, this knowledge can aid healthcare providers in creating more effective screening tools to guide subsequent treatment for those struggling with addiction [73]. Identifying personality variables that serve as risk factors for addiction may contribute to a deeper understanding of addiction and also aid its prevention and treatment, as it highlights key personality traits that should be addressed in such interventions. Understanding which personality traits predispose individuals to these disorders can inform the development of personalized treatment plans, making interventions more effective by addressing specific personality-related vulnerabilities. For instance, individuals with high levels of certain traits may benefit from tailored therapeutic approaches such as cognitive-behavioral therapy or motivational interviewing. Additionally, the identification of personality traits that serve as risk factors for addiction can aid in creating preventive strategies, with educational programs and community interventions designed to target these traits and reduce the incidence of addiction by fostering resilience and promoting healthier coping mechanisms.

Given the limited number of studies on the association between HEXACO personality traits and SUD, we suggest that future research should focus on exploring HEXACO traits in relation to various types of SUD, including alcohol, cannabis, and other narcotics. Longitudinal studies that can inform about the directionality between the personality traits and various addictions as well as the HEXACO personality traits as moderators of addiction treatment effects would be of special interest. Additionally, more research is needed on other types of behavioral addictions, such as gaming problems, CSBD, and social media addiction, and how these relate to the HEXACO traits. Researchers should also aim to provide more detailed information on demographic factors like gender, age, and geographical location in their studies to enhance the understanding of these associations and improve the generalizability of findings. Moreover, future studies should consider conducting research on different sample types, such as adolescents, elderly populations, individuals with co-occurring mental health disorders, and diverse cultural backgrounds, to explore how personality traits contribute to addiction risk across various populations.

The present study has several strengths. As the first systematic review and meta-analysis in this field, our study will fill a significant gap in the literature by providing a comprehensive overview of the associations between HEXACO personality traits, SUD, and behavioral addictions. It will be conducted in line with the PRISMA guidelines [74]. We will use the Covidence Systematic Review software for data management throughout the systematic review process to manage references, which can increase reliability and ease collaboration between reviewers [67]. Searches will be conducted across several databases, and we will utilize additional methods in our literature search, including artificial intelligence using Keenious Plus. To ensure reliability regarding quality assessment and effect size data, the included studies will be coded independently by two authors, with disagreements being resolved by discussion or referral to a third reviewer. Moreover, a pilot screening process was conducted to ensure consistency and reliability, and a pilot test regarding data extraction was carried out to assess feasibility and refine the data extraction form. The results of these pilot tests showed a high level of inter-reviewer agreement.

While this study has several strengths, the results of this systematic review should be interpreted with caution due to several potential limitations. One significant limitation is the scarcity of existing research specifically focused on the associations between the HEXACO model, SUD, and behavioral addictions. The HEXACO model is relatively new compared to other personality frameworks, such as the FFM, and therefore, the number of studies investigating its relationship with addictive disorders may be limited. This scarcity can constrain the pool of eligible studies for inclusion in a systematic review/meta-analysis, potentially affecting the robustness and generalizability of the findings. Additionally, our initial search indicated that there are fewer studies related to HEXACO personality traits and SUD compared to those focused on HEXACO and behavioral addictions, which may impact the comprehensiveness of our results. Furthermore, studies that have not reported correlation coefficients or coefficients convertible to correlation coefficients cannot be included in this meta-analysis, which may lead to the exclusion of relevant research. Moreover, not all studies may report key variables such as age, gender, ethnicity, and marital status, which are considered potential moderators that can help explain variations in effect sizes. It is also significant to note that grey literature was not incorporated into the research for this study. The inclusion of grey literature could have provided a more comprehensive view of the topic by incorporating unpublished research and findings that might not be accessible through conventional academic publishing channels.

Despite the limitations, this systematic review meta-analysis study will help clarify the role of HEXACO personality traits underlying addiction among the studies conducted to date. The results will have implications for understanding the role of personality traits in addiction. Identifying specific HEXACO traits linked to SUD and behavioral addictions will improve screening, prevention, and treatment strategies tailored to individual personality profiles.

## Supplementary Information

Additional file 1. PRISMA-P 2015 Checklist.

Additional file 2. Extraction Sheet.

## Data Availability

Data sharing is not applicable to this article as no datasets were generated or analyzed during the current study.

## Abbreviations

SUD: Substance use disorders
PRISMA-P: Preferred Reporting Items for Systematic Review and Meta-Analysis Protocols
NOS: Newcastle-Ottawa Scale
CMA: Comprehensive meta-analysis
CSBD: Compulsive sexual behavior disorder
FFM: Five-Factor Model
AUD: Alcohol use disorder
GD: Gambling disorder
CUD: Cannabis use disorder
PGSI: Problem Gambling Severity Index
PROSPERO: International Prospective Register of Systematic Reviews

## References

[1] Rachlin H. Why do people gamble and keep gambling despite heavy losses? Psychol Sci. 1990;1(5):294–6. 10.1111/j.1467-9280.1990.tb00220.x.

[2] Zeng X, Han X, Zheng D, Jiang P, Yuan Z. Similarity and difference in large-scale functional network alternations between behavioral addictions and substance use disorder: a comparative meta-analysis. Psychol Med. 2024;54(3):473–87. 10.1017/S0033291723003434. 38047402 10.1017/S0033291723003434

[3] James SL, Abate D, Abate KH, Abay SM, Abbafati C, Abbasi N, et al. Global, regional, and national incidence, prevalence, and years lived with disability for 354 diseases and injuries for 195 countries and territories, 1990–2017: a systematic analysis for the Global Burden of Disease Study 2017. The Lancet. 2018;392(10159):1789–858. 10.1016/S0140-6736(18)32279-7. 10.1016/S0140-6736(18)32279-7 PMC6227754 30496104

[4] Jaguga F, Kiburi SK, Temet E, Barasa J, Karanja S, Kinyua L, et al. A systematic review of substance use and substance use disorder research in Kenya. PLoS ONE. 2022;17(6):1–54. 10.1371/journal.pone.0269340. 10.1371/journal.pone.0269340 PMC9186181 35679248

[5] O’Shea B. Diagnostic and statistical manual of mental disorders (revised). American Psychiatric Association. Washington, DC: APA 1987. Pp 567. Ir J Psychol Med. 1989;6(1):54. 10.1017/S0790966700015767.

[6] Organization WH. The ICD-10 classification of mental and behavioural disorders: diagnostic criteria for research: World Health Organization; 1993. 10.1177/146642409311300216.

[7] Griffiths M. Behavioural addiction: an issue for everybody? Employee Councelling Today. 1996;8(3):19–25. 10.1108/13665629610116872.

[8] Marks I. Behavioural (non-chemical) addictions. Br J Addict. 1990;85(11):1389–94. 10.1111/j.1360-0443.1990.tb01618.x. 2285832 10.1111/j.1360-0443.1990.tb01618.x

[9] Andreassen CS, Hetland J, Pallesen S. The relationship between ‘workaholism’, basic needs satisfaction at work and personality. Eur J Pers. 2010;24(1):3–17. 10.1002/per.737.

[10] Fisher S. Identifying video game addiction in children and adolescents. Addict Behav. 1994;19(5):545–53. 10.1016/0306-4603(94)90010-8. 7832013 10.1016/0306-4603(94)90010-8

[11] OReilly M. Internet addiction: a new disorder enters the medical lexicon. CMAJ. 1996;154(12):1882–3 https://bit.ly/3sgLX5V. 8653648 PMC1487729

[12] Griffiths M. Exercise addiction: a case study. Addict Res. 1997;5(2):161–8. 10.3109/16066359709005257.

[13] Chóliz M. Mobile phone addiction: a point of issue. Addiction. 2010;105(2):373–4. 10.1111/j.1360-0443.2009.02854.x. 20078493 10.1111/j.1360-0443.2009.02854.x

[14] Christenson GA, Faber RJ, De Zwaan M, Raymond NC, Specker SM, Ekern MD, et al. Compulsive buying: descriptive characteristics and psychiatric comorbidity. J Clin Psychiatry. 1994;55(1):5–11. https://pubmed.ncbi.nlm.nih.gov/8294395/. 8294395

[15] Kraus SW, Voon V, Potenza MN. Should compulsive sexual behavior be considered an addiction? Addiction. 2016;111(12):2097–106. 10.1111/add.13297. 26893127 10.1111/add.13297 PMC4990495

[16] Krueger RB. Diagnosis of hypersexual or compulsive sexual behavior can be made using ICD-10 and DSM-5 despite rejection of this diagnosis by the American Psychiatric Association. 2016; 2010–2111. 10.1111/add.13366. 10.1111/add.13366 27086656

[17] Pan Y-C, Chiu Y-C, Lin Y-H. Systematic review and meta-analysis of epidemiology of internet addiction. Neurosci Biobehav Rev. 2020;118:612–22. 10.1016/j.neubiorev.2020.08.013. 32853626 10.1016/j.neubiorev.2020.08.013

[18] Çakıcı M, Çakıcı E, Babayiğit A, Karaaziz M. Gambling behaviour: prevalence, risk factors and relation with acculturation in 2007–2018 North Cyprus adult household surveys. J Gambl Stud. 2021;37(4):1099–111. 10.1007/s10899-021-10008-3. 33515175 10.1007/s10899-021-10008-3

[19] Leshner AI. Addiction is a brain disease, and it matters. Science. 1997;278(5335):45–7. 10.1126/science.278.5335.45. 9311924 10.1126/science.278.5335.45

[20] Moran P, Coffey C, Mann A, Carlin JB, Patton GC. Personality and substance use disorders in young adults. Br J Psychiatry. 2006;188(4):374–9. 10.1192/bjp.188.4.374. 16582065 10.1192/bjp.188.4.374

[21] Walker MB. Some problems with the concept of “gambling addiction”: should theories of addiction be generalized to include excessive gambling? J Gambl Behav. 1989;5:179–200. 10.1007/bf01024386.

[22] Dick DM, Aliev F, Latendresse SJ, Hickman M, Heron J, Macleod J, et al. Adolescent alcohol use is predicted by childhood temperament factors before age 5, with mediation through personality and peers. Alcohol Clin Exp Res. 2013;37(12):2108–17. 10.1111/acer.12206. 23841856 10.1111/acer.12206 PMC3823677

[23] Wallace A, Ullsperger JM, Nikolas MA. Do personality traits explain the association between childhood attention-deficit hyperactivity disorder symptoms and substance use and problems in young adults? Personality Individ Differ. 2016;92:22–8. 10.1016/j.paid.2015.11.046.

[24] Goldberg LR. The structure of phenotypic personality traits. Am Psychol. 1993;48(1):26–34. 10.1037//0003-066x.48.1.26. 8427480

[25] Hyman SE. The Science of Mental Health: Compulsive Disorder and Tourette's Syndrome: Taylor & Francis US; 2001. 10.4324/9780203822937.

[26] Grekin ER, Sher KJ, Wood PK. Personality and substance dependence symptoms: modeling substance-specific traits. Psychol Addict Behav. 2006;20(4):415–24. 10.1037/0893-164x.20.4.415. 17176176 10.1037/0893-164X.20.4.415

[27] Bagby RM, Costa PT Jr, McCrae RR, Livesley WJ, Kennedy SH, Levitan RD, et al. Replicating the five factor model of personality in a psychiatric sample. Personality Individ Differ. 1999;27(6):1135–9. 10.1016/s0191-8869(99)00055-0.

[28] Kircaburun K, Griffiths MD. Instagram addiction and the Big Five of personality: The mediating role of self-liking. J Behav Addict. 2018;7(1):158–70. 10.1556/2006.7.2018.15. 29461086 10.1556/2006.7.2018.15 PMC6035031

[29] Kim Y, Jeong JE, Cho H, Jung DJ, Kwak M, Rho MJ, et al. Personality factors predicting smartphone addiction predisposition: behavioral inhibition and activation systems, impulsivity, and self-control. PLoS ONE. 2016;11(8):1–15. 10.1371/journal.pone.0159788. 10.1371/journal.pone.0159788 PMC4988723 27533112

[30] Amichai-Hamburger Y, Vinitzky G. Social network use and personality. Comput Hum Behav. 2010;26(6):1289–95. 10.1016/j.chb.2010.03.018.

[31] Kim EJ, Namkoong K, Ku T, Kim SJ. The relationship between online game addiction and aggression, self-control and narcissistic personality traits. Eur Psychiatry. 2008;23(3):212–8. 10.1016/j.eurpsy.2007.10.010. 18166402 10.1016/j.eurpsy.2007.10.010

[32] Pinto J, Carvalho J, Nobre PJ. The relationship between the FFM personality traits, state psychopathology, and sexual compulsivity in a sample of male college students. J Sex Med. 2013;10(7):1773–82. 10.1111/jsm.12185. 23815239 10.1111/jsm.12185

[33] Engel J, Carstensen M, Veit M, Sinke C, Kneer J, Hartmann U, et al. Personality dimensions of compulsive sexual behavior in the Sex@ Brain study. J Behav Addict. 2023;12(2):408–20. 10.1556/2006.2023.00029. 37384566 10.1556/2006.2023.00029 PMC10316167

[34] Terracciano A, Löckenhoff CE, Crum RM, Bienvenu OJ, Costa PT. Five-Factor Model personality profiles of drug users. BMC Psychiatry. 2008;8(1):1–10. 10.1186/1471-244x-8-22. 18405382 10.1186/1471-244X-8-22 PMC2373294

[35] Malouff JM, Thorsteinsson EB, Rooke SE, Schutte NS. Alcohol involvement and the five-factor model of personality: a meta-analysis. J Drug Educ. 2007;37(3):227–94. 10.2190/de.37.3.d. 18047183 10.2190/DE.37.3.d

[36] Malouff JM, Thorsteinsson EB, Schutte NS. The five-factor model of personality and smoking: a meta-analysis. J Drug Educ. 2006;36(1):47–58. 10.2190/9ep8-17p8-ekg7-66ad. 16981639 10.2190/9EP8-17P8-EKG7-66AD

[37] Ashton MC, Lee K, Goldberg LR. A hierarchical analysis of 1,710 English personality-descriptive adjectives. J Pers Soc Psychol. 2004;87(5):707–21. 10.1037/0022-3514.87.5.707. 15535781 10.1037/0022-3514.87.5.707

[38] Ashton MC, Lee K, De Vries RE. The HEXACO honesty-humility, agreeableness, and emotionality factors: a review of research and theory. Pers Soc Psychol Rev. 2014;18(2):139–52. 10.1177/1088868314523838. 24577101 10.1177/1088868314523838

[39] Ashton MC, Lee K. The HEXACO model of personality structure. In: The SAGE Handbook of Personality Theory and Assessment. 2008;2:239-260. 10.4135/9781849200479.n12.

[40] Ashton MC, Lee K. Empirical, theoretical, and practical advantages of the HEXACO model of personality structure. Pers Soc Psychol Rev. 2007;11(2):150–66. 10.1177/1088868306294907. 18453460 10.1177/1088868306294907

[41] Lee K, Ashton MC. The HEXACO personality factors in the indigenous personality lexicons of English and 11 other languages. J Pers. 2008;76(5):1001–54. 10.1111/j.1467-6494.2008.00512.x. 18665898 10.1111/j.1467-6494.2008.00512.x

[42] John OP, Naumann LP, Soto CJ. Paradigm shift to the integrative big five trait taxonomy. Handbook of personality: Theory and research. 2008;3(2):114–158. https://bit.ly/3EzwAtQ.

[43] Aghababaei N, Mohammadtabar S, Saffarinia M. Dirty Dozen vs. the H factor: Comparison of the Dark Triad and Honesty-Humility in prosociality, religiosity, and happiness. Pers Individ Differ. 2014;67:6–10. 10.1016/j.paid.2014.03.026.

[44] Bresin K, Gordon KH. Characterizing pathological narcissism in terms of the HEXACO model of personality. J Psychopathol Behav Assess. 2011;33:228–35. 10.1007/s10862-010-9210-9.

[45] De Vries RE, van Kampen D. The HEXACO and 5DPT models of personality: A comparison and their relationships with psychopathy, egoism, pretentiousness, immorality, and machiavellianism. J Pers Disord. 2010;24(2):244–57. 10.1521/pedi.2010.24.2.244. 20420478 10.1521/pedi.2010.24.2.244

[46] Djeriouat H, Trémolière B. The dark triad of personality and utilitarian moral judgment: The mediating role of honesty/humility and harm/care. Personality Individ Differ. 2014;67:11–6. 10.1016/j.paid.2013.12.026.

[47] Hodson G, Book A, Visser BA, Volk AA, Ashton MC, Lee K. Is the dark triad common factor distinct from low honesty-humility? J Res Pers. 2018;73:123–9. 10.1016/j.jrp.2017.11.012.

[48] De Vries A, de Vries RE, Born MP. Broad versus narrow traits: conscientiousness and honesty–humility as predictors of academic criteria. Eur J Pers. 2011;25(5):336–48. 10.1002/per.795.

[49] Kim HS, Rash CL, McGrath DS. The dishonest gambler: low HEXACO honesty–humility and gambling severity in a community sample of gamblers. Personal Ment Health. 2018;12(4):335–64. 10.1002/pmh.1433. 10.1002/pmh.1433 30203924

[50] Leslie RD, McGrath DS. A comparative Profile of Online, Offline, and Mixed-Mode problematic gamblers’ gambling involvement, motives, and HEXACO personality traits. J Gambl Stud. 2023:1–17. 10.1007/s10899-023-10193-3. 10.1007/s10899-023-10193-3 PMC9897996 36737531

[51] McGrath DS, Neilson T, Lee K, Rash CL, Rad M. Associations between the HEXACO model of personality and gambling involvement, motivations to gamble, and gambling severity in young adult gamblers. J Behav Addict. 2018;7(2):392–400. 10.1556/2006.7.2018.29. 29846096 10.1556/2006.7.2018.29 PMC6174587

[52] Rash CL, An examination of the HEXACO model of personality in alcohol use disorder, cannabis use disorder, and gambling disorder [Master's thesis]. Calgary: University of Calgary; 2018: Arts. https://bit.ly/3Lr8pXb.

[53] Johnson MK, Rowatt WC, Petrini L. A new trait on the market: honesty–humility as a unique predictor of job performance ratings. Personality Individ Differ. 2011;50(6):857–62. 10.1016/j.paid.2011.01.011.

[54] Zettler I, Hilbig BE, Heydasch T. Two sides of one coin: honesty–humility and situational factors mutually shape social dilemma decision making. J Res Pers. 2013;47(4):286–95. 10.1016/j.jrp.2013.01.012.

[55] Abbasi M, Hajilou N, Atadokht A, Narimani M. The relationship between addication to cell phones and religious attitude, psychological wellbeing and personality attribute [Master's thesis]. Ardabil: University of Mohaghegh Ardabili; 2014. https://bit.ly/3Mc6mO5.

[56] Enayati S. Predicting mobile phone addiction based on Hexaco personality traits, cognitive flexibility and time perspective in female students. 5(48);1–18. https://bit.ly/3MwOYg1.

[57] Azizi A, Esmaeli S, Esmaeli M, Peyda N. Internet addiction in adolescents with HEXACO personality trait-A correlational study. J Nurs Educ. 2015;4(2):68–77 http://jne.ir/article-1-487-en.html.

[58] Horwood S, Anglim J. Personality and problematic smartphone use: a facet-level analysis using the five factor model and HEXACO frameworks. Comput Hum Behav. 2018;85:349–59. 10.1016/j.chb.2018.04.013.

[59] Inanloo M, Bashardoust S, Alhosseini KA. Providing a model for predicting online game addiction based on HEXACO personality traits and the parent-child relationship mediated by impulsiveness. Appl Fam Ther J. 2022;3(1):285–310. 10.61838/kman.aftj.3.1.15.

[60] Zafar M, Lodhi IS, Shakir M. Impact of personality traits on facebook addiction: the mediating role of perceived social support. J Res Soc Sci. 2018;6(1):239–58 https://bit.ly/3LR85M8.

[61] Horwood S, Anglim J. Self and other ratings of problematic smartphone use: The role of personality and relationship type. Comput Hum Behav. 2021;116:1–10. 10.1016/j.chb.2020.106634.

[62] Borenstein M, Hedges LV, Higgins JP, Rothstein HR. Introduction to meta-analysis: John Wiley & Sons; 2021. 10.1002/9781119558378.

[63] Booth A, Clarke M, Dooley G, Ghersi D, Moher D, Petticrew M, et al. The nuts and bolts of PROSPERO: an international prospective register of systematic reviews. Syst Rev. 2012;1(1):1–9. 10.1186/2046-4053-1-2. 22587842 10.1186/2046-4053-1-2 PMC3348673

[64] Shamseer L, Moher D, Clarke M, Ghersi D, Liberati A, Petticrew M, et al. Preferred reporting items for systematic review and meta-analysis protocols (PRISMA-P) 2015: elaboration and explanation. BMJ. 2015;349:1–25. 10.1136/bmj.g7647. 10.1136/bmj.g7647 25555855

[65] Moher D, Liberati A, Tetzlaff J, Altman DG, Group P. Preferred reporting items for systematic reviews and meta-analyses: the PRISMA statement. Int J Surg. 2010;8(5):336–41336–341. 10.1016/j.ijsu.2010.02.007. 10.1016/j.ijsu.2010.02.007 20171303

[66] McHugh ML. Interrater reliability: the kappa statistic. Biochem Med (Zagreb). 2012;22(3):276–82 https://bit.ly/3LRN4rK. 23092060 PMC3900052

[67] Macdonald M, Misener RM, Weeks L, Helwig M. Covidence vs Excel for the title and abstract review stage of a systematic review. JBI Evidence Implementation. 2016;14(4):200–1. 10.1097/01.xeb.0000511346.12446.f2.

[68] Higgins JP, Altman DG, Gøtzsche PC, Jüni P, Moher D, Oxman AD, et al. The cochrane collaboration’s tool for assessing risk of bias in randomised trials. Br Med J. 2011;1–9. 10.1136/bmj.d5928. 10.1136/bmj.d5928 PMC3196245 22008217

[69] Wells GA, Shea B, O’Connell D, Peterson J, Welch V, Losos M, et al. The Newcastle-Ottawa Scale (NOS) for assessing the quality of nonrandomised studies in meta-analyses. 2000:1–12. https://bit.ly/3QvWflQ.

[70] Hedges LV, Vevea JL. Fixed-and random-effects models in meta-analysis. Psychol Methods. 1998;3(4):1–19. 10.1037//1082-989x.3.4.486.

[71] Higgins JP, Thompson SG, Deeks JJ, Altman DG. Measuring inconsistency in meta-analyses. Br Med J. 2003;327(7414):557–60. 10.1136/bmj.327.7414.557. 10.1136/bmj.327.7414.557 PMC192859 12958120

[72] Borenstein M. Comprehensive meta‐analysis software. Systematic reviews in health research: meta‐analysis in context. 2022:535–548. 10.1002/9781119099369.ch27.

[73] Błachnio A, Przepiorka A, Pantic I. Association between Facebook addiction, self-esteem and life satisfaction: A cross-sectional study. Comput Hum Behav. 2016;55:701–5. 10.1016/j.chb.2015.10.026.

[74] Moher D, Liberati A, Tetzlaff J, Altman DG, Grp P. Preferred Reporting Items for Systematic Reviews and Meta-Analyses: The PRISMA Statement (Reprinted from Annals of Internal Medicine). Phys Ther. 2009;89(9):873–80. 10.1093/ptj/89.9.873. 19723669

