# Supplementary material for: Associations between HEXACO personality traits, substance use disorders, and behavioral addictions: a protocol for a comprehensive systematic review and meta-analysis
Source: Syst Rev. 2025 Jan 2;14:1. doi: 10.1186/s13643-024-02741-8 (PMC11694390; doi:10.1186/s13643-024-02741-8)
Supplement: Supplementary file 2 — Additional file 2. Extraction Sheet. [file 13643_2024_2741_MOESM2_ESM.docx]

**Extraction Sheet**

| **Variable name** | **Codes** | **Definition/Instructions & examples** |
| --- | --- | --- |
| **Report identification** | | |
| Coder ID | 1: FSH  2: PH | State the coder ID (e.g., FSH: 1, PH: 2). |
| Study DOI | = | State the DOI of the paper/dissertation. |
| Title | = | State the full title of the paper/dissertation (e.g., The dishonest gambler: Low HEXACO honesty–humility and gambling severity in a community sample of gamblers). |
| Author(s), Year | = | State the last name of the first author and the year of publication (e.g., Reyes et al. (2019)). |
| Journal | = | State the full name of the journal (e.g., Current Psychology). |
| Language | 1: English  2: Persian  3: Other; specify | Select the languages of the paper/dissertation. |
| Publication type | 1: Research paper  2: Dissertation/thesis | 1: Primary articles published in peer-reviewed journals  2: PhD/MA – theses. |
| **Study setting** | | |
| Study goal | = | State the goals of the paper/dissertation (e.g., assessing whether the HEXACO dimensions are associated with gambling engagement and gambling severity). |
| Study design | 1: Cross-sectional study  2: longitudinal study  3: Experimental/Intervention study (e.g., RCT)  4: Case-control study | Select the appropriate description of study design. |
| Country | = | State the country of origin for the paper/dissertation and participants (e.g., Norway). If several countries, state all. If unknown, mark as missing. |
| Start of data collection (month, year) | = | State the month and year the data collection started and ended (e.g., January 2020 – January 2021). |
| - **If 2:** Longitudinal study; duration of follow-up (months) | = | State the duration of the follow-up period in months. If multiple timepoints, state all periods (e.g., 6 months, 8, months). |
| Ethical approval | 1: yes, have received approval  2: No / not stated | State whether the study has received ethical approval (e.g., from a university or regional ethics committee). |
| Conflict of interest statement | 1: Yes, no conflicts  2: No, no declaration available  3: Yes, conflicts stated | State whether the study declared a conflict of interest statement or not. |
| **Participants** | | |
| Sample type | 1: High school students  2: University students (Undergraduate)  3: University student (Graduate)  4: General Population Sample  5: Participants with substance use disorders  6: Participants with behavioral addictions  7: Mix sample type  8: Other |  |
| - **If 5:** Participants with substance use disorders; specify - **If 6:** Participants with behavioral addictions; specify - **If 7:** Mix sample type; specify - **If 8:** Other; specify | = | - (e.g., Participants with alcohol use disorder) - (e.g., Participants with internet addiction) - (e.g., Participants with alcohol use disorder+ internet addiction). - If none of the above; specify. |
| Total sample size (n) | = | State the total amount of participants in the study (e.g., that was included in the analyses). |
| Sample size participants with substance use disorders/ behavioral addictions. | = | State the total amount of participants with substance use disorders/ behavioral addictions in the study (e.g., that was included in the analyses). |
| Sample size control group | = | State the total amount of participants in control group in the study (e.g., that was included in the analyses). |
| Total age range | = | State the total age range of the sample population. |
| Mean age reported | = | State the overall mean age of the sample population. |
| Gender distribution | 1: Male  2: Female  3: Other/Non-binary  4: Mix | Specify the sex distribution of the sample population. |
| - **If 4:** Mix; specify |  |  |
| Total Sex (% men/women) | = | State the percentage of men/women/non-binary in whole sample. |
| Type of selection | 1: Simple random sampling  2: Cluster random sampling  High school / College / University students  3: Stratified sampling  4: Convenience sampling  5: Substance use disorders/ behavioral addictions community  6: Medical records/Register data  7: Treatment  8: Other | 1: Selection is based on random population sampling  2: Selection is based on cluster random sampling  3: Selection is based on stratified sampling  4: Selection is based on convenience sampling  5: Selection is recruited through substance use disorders/ behavioral addictions community (e.g., Alcoholics Anonymous (AA), Narcotics Anonymous (NA), Gamblers Anonymous (GA), and other related communities)  6: Selection (and data) is based individuals registered in medical records/register data  7: Selection is based on a sample actively receiving or seeking treatment  8: Other: If none of the above; specify. |
| - **If 8:** Other; specify |  |  |
| Comorbid mental health disorders     - **If 5:** Mix; specify - **If 6:** Other; specify | 1: Depression  2: Anxiety  3: Personality disorder(s)  4: Bipolar disorder  5: Mix disorders  6: Other | Select all comorbid mental health disorders reported within the SUD and behavioral addictions sample. Comorbidity must be above a clinical threshold assessed by a clinician or by conventional cut-off in standardized/validated measures indicating (probable). The outcome must be categorical (i.e., yes/no). Generic/mean scores on e.g., a depression scale without categorization (e.g., “mean depression is not to be coded.  5: Mix; specify  6: Other: If none of the above; specify. |
| Comorbidity assessed instrument | = | State the instrument used for assessing comorbidity in the sample. |
| Comorbidity reported % of sample | = | State the percentage of comorbidity in the SUD and behavioral addictions sample. Use classifications above (e.g., Depression: 34%). |
| **Methodology** | | |
| HEXACO personality inventory- revised (HEXACO-PI-R) instrument | 1: HEXACO-24  2: HEXACO-60  3: HEXACO-100  4: HEXACO-200  5: Other | Select the version of HEXACO-PI-R instrument used in assessing personality traits. |
| - **If 5:** Other; specify |  |  |
| Included personality traits | 1: All six traits included  2. Other; specify | 1: All six traits were assessed, including Honesty-Humility, Emotionality, Extraversion, Agreeableness, Conscientiousness, and Openness to Experience.  2: Other; only some of the personality traits were assessed; specify (e.g., Honesty-Humility and Emotionality). |
| Type of substance use disorders assessed | 1: Alcohol use  2: Nicotine use  3: Cannabis use  4: Narcotics use  5: Other; specify | Select the type of SUD assessed among participant. |
| - **If 2:** Nicotine use; specify - **If 3:** Cannabis use; specify - **If 4:** Narcotics use; specify - **If 5:** Other; specify | = | - State the type of nicotine use assessed among participant - State the type of cannabis use assessed among participant - State the type of narcotics use assessed among participant - If none of the above; specify. |
| Substance use disorders data collection /assessment method | 1: Self-report measure  2: Clinical interview  3: Objective measurements  4: Psychological autopsy  5: Self-categorization  6: Mix method  7: Other | 1: Self-reported measurements (i.e., questionnaires/survey, validated scale or single item)  2: Assessment and categorization by clinician through diagnostic interview  3: Objective measurements (e.g., player tracking data, health registry)  4: Collection of information on the deceased / assessment via structured interviews of family members, relatives, friends, health care personnel  5: Self-categorization/diagnosis based on the individual’s own perception/assessment  6: If mix method used; specify  7: If none of the above; specify. |
| - **If 6:** Mix method; specify - **If 7:** Other; specify |  |  |
| Substance use disorders instrument | = | State the name of the instrument used in assessing SUD as reported by the paper/dissertation. If there is a single item, write the whole question. |
| SUD conceptualization method | 1: Categorical variable  2: Continuous (Yes/No) | 1: Categorical variable: Specify if addiction is measured on a categorical variable and, if so, in the next step you should provide the cutoff point if applicable (e.g., addiction severity was measured using the Addiction Severity Index (ASI), with a cutoff score of 20 indicating clinically significant addiction.).  2: Continuous: Indicate whether the study defines addiction as a continuous (e.g., presence or absence of addiction)(e.g., addiction was defined as the presence or absence of substance use disorder based on DSM-5 criteria). |
| - **If 2:** Categorical variable; specify cutoff point if applicable | = |  |
| Method of conceptualization | 1: Time spent on the activity (e.g., hours/day, days/week)  2: Frequency of engagement (e.g., episodes/week, times/day)  3: Severity of symptoms (e.g., mild, moderate, severe)  4: Number of symptoms present  5: Diagnostic criteria (e.g., DSM-5, ICD-11)  6: Self-reported addiction (Yes/No)  7: Clinical diagnosis (Yes/No)  8: Mix method  9: Other | 1: Time spent on the activity: Measure addiction based on the amount of time spent engaging in the activity (e.g., hours/day, days/week).  2: Frequency of engagement: Assess addiction based on the frequency of engaging in the activity (e.g., episodes/week, times/day).  3: Severity of symptoms: Define addiction severity based on the intensity or severity of symptoms experienced (e.g., mild, moderate, severe).  4: Number of symptoms present: Determine addiction based on the number of symptoms present according to established criteria (e.g., DSM-5, ICD-11).  5: Diagnostic criteria: Use standardized diagnostic criteria such as DSM-5 or ICD-11 to diagnose addiction.  6: Self-reported addiction: Rely on self-report measures to assess addiction (Yes/No).  7: Clinical diagnosis: Determine addiction through clinical assessment and diagnosis (Yes/No).  8: Mix method: Specify  9: Other: Specify any additional method used by the study to conceptualize addiction. |
| - **If 8:** Mix method; specify - **If 9:** Other; specify |  |  |
| Type of behavioral addiction assessed | 1: Gambling  2: Gaming  3: Mobile phone addiction  4: Internet addiction  5: Facebook addiction  6: Exercise addiction  7: Compulsive buying  8: Study addiction  9: Mix behavioral addiction  10: Other | Select the type of behavioral addictions assessed among participants |
| - **If 9:** Mix behavioral addiction; specify - **If 9:** Other; specify |  |  |
| Behavioral addictions data collection /assessment method | 1: Self-report measure  2: Clinical interview  3: Objective measurements  4: Psychological autopsy  5: Self-categorization  6: Mix method  7: Other | 1: Self-reported measurements (i.e., questionnaires/survey, validated scale, or single item)  2: Assessment and categorization by clinician through diagnostic interview  3: Objective measurements (e.g., player tracking data, health registry)  4: Collection of information on the deceased / assessment via structured interviews of family members, relatives, friends, health care personnel  5: Self-categorization/diagnosis based on the individual’s own perception/assessment  6: If mix method used; specify  7: If none of the above; specify. |
| - **If 6:** Mix method; specify - **If 7:** Other; specify |  |  |
| Behavioral addictions instrument | = | State the name of the instrument used in assessing behavioral addictions as reported by the paper/dissertation. If single item, write the whole question |
| Behavioral addictions conceptualization method | 1: Categorical variable  2: Continuous (Yes/No) | 1: Categorical variable: Specify if addiction is measured on a categorical variable and, if so, in the next step you should provide the cutoff point if applicable (e.g., addiction severity was measured using the Addiction Severity Index (ASI), with a cutoff score of 20 indicating clinically significant addiction.).  2: Continuous: Indicate whether the study defines addiction as a continuous (e.g., presence or absence of addiction)(e.g., addiction was defined as the presence or absence of substance use disorder based on DSM-5 criteria). |
| - **If 2:** Categorical variable; specify cutoff point if applicable | = |  |
| Method of conceptualization | 1: Time spent on the activity (e.g., hours/day, days/week)  2: Frequency of engagement (e.g., episodes/week, times/day)  3: Severity of symptoms (e.g., mild, moderate, severe)  4: Number of symptoms present  5: Diagnostic criteria (e.g., DSM-5, ICD-11)  6: Self-reported addiction (Yes/No)  7: Clinical diagnosis (Yes/No)  8: Mix method  9: Other | 1: Time spent on the activity: Measure addiction based on the amount of time spent engaging in the activity (e.g., hours/day, days/week).  2: Frequency of engagement: Assess addiction based on the frequency of engaging in the activity (e.g., episodes/week, times/day).  3: Severity of symptoms: Define addiction severity based on the intensity or severity of symptoms experienced (e.g., mild, moderate, severe).  4: Number of symptoms present: Determine addiction based on the number of symptoms present according to established criteria (e.g., DSM-5, ICD-11).  5: Diagnostic criteria: Use standardized diagnostic criteria such as DSM-5 or ICD-11 to diagnose addiction.  6: Self-reported addiction: Rely on self-report measures to assess addiction (Yes/No).  7: Clinical diagnosis: Determine addiction through clinical assessment and diagnosis (Yes/No).  8: Mix method: Specify  9: Other: Specify any additional method used by the study to conceptualize addiction. |
| Statistical analysis used |  | State the method of analysis used. |
| **Results** | | |
| Effect size reported | 1: Pearson’s correlation  2: Spearman’s correlation  3: Other; specify | Select the type of effect size reported in this paper/dissertation. |
| - **If 3:** Other; specify |  |  |
| Effect size (r) | = | State the percentage (%) of correlation between HEXACO personality traits, substance use disorders, and behavioral addiction. |
| - ***If the (r) is not provided*** ***directly,*** sample size (N), standard deviation (SD), and means for both groups | - *Addiction group:*   1: Sample size (N): (%)  2: Standard deviation (SD): (%)  3: Means: (%)  4: Converted correlation coefficient (r): (%)   - *Control group:*   1: Sample size (N): (%)  2: Standard deviation (SD): (%)  3: Means: (%)  4: Converted correlation coefficient (r) | State the percentage (%) of sample size (N), standard deviation (SD), and means for both the addiction and control groups in the paper/dissertation. |
| - **If the (r) is not provided directly,** converted into a correlation coefficient based on Cohen’s d/f, T value, or Fishers Z for both groups | - *Addiction group:*   1: Cohen’s d/f: (%)  2: T value: (%)  3: Fishers Z: (%)  4: Converted correlation coefficient (r):   - *Control group:*   1: Cohen’s d/f: (%)  2: T value: (%)  3: Fishers Z: (%)  4: Converted correlation coefficient (r) | State the percentage (%) of Cohen’s d/f, T value, or Fisher's Z for converted data into a correlation coefficient for both the addiction and control groups in the paper/dissertation. |
| Main finding |  | For example: Gaming addiction was negatively correlated with extraversion and conscientiousness. |
| Study limitations |  | For example: Self-selected sample; limited generalizability because the data were collected from only MMOGs players. |
